# Cost of postoperative complications of lower anterior resection for rectal cancer: a nationwide registry study of 15,187 patients

**DOI:** 10.1007/s00595-022-02523-6

**Published:** 2022-05-24

**Authors:** Hiraku Kumamaru, Yoshihiro Kakeji, Kiyohide Fushimi, Koichi Benjamin Ishikawa, Hiroyuki Yamamoto, Hideki Hashimoto, Minoru Ono, Tadashi Iwanaka, Shigeru Marubashi, Mitsukazu Gotoh, Yasuyuki Seto, Yuko Kitagawa, Hiroaki Miyata

**Affiliations:** 1 Department of Healthcare Quality Assessment, The University of Tokyo Graduate School of Medicine, 7-3-1 University of Tokyo Hospital Chuoushinryoutou II, 8F, Hongo, Tokyo, 113-8655 Japan; 2 Database Committee, The Japanese Society of Gastroenterological Surgery, Tokyo, Japan; 3 Department of Health Policy and Informatics, Tokyo Medical and Dental University, Tokyo, Japan; 4 International University of Health and Welfare, Tokyo, Japan; 5 Department of Health and Social Behavior, The University of Tokyo School of Public Health, Tokyo, Japan; 6 Department of Cardiovascular Surgery, The University of Tokyo Graduate School of Medicine, Tokyo, Japan; 7 The Japanese Society of Gastroenterological Surgery, Tokyo, Japan

**Keywords:** Clavien–Dindo classification, Cost, Lower anterior resection, Postoperative complications

## Abstract

**Purpose:**

To assess the increase in hospital costs associated with postoperative complications after lower anterior resection (LAR) for rectal cancer.

**Methods:**

The subjects of this retrospective analysis were patients who underwent elective LAR surgery between April, 2015 and March, 2017, collected from a Japanese nationwide gastroenterological surgery registry linked to hospital-based claims data. We evaluated total and category-specific hospitalization costs based on the level of postoperative complications categorized using the Clavien–Dindo (CD) classification. We assessed the relative increase in hospital costs, adjusting for preoperative factors and hospital case volume.

**Results:**

We identified 15,187 patients (mean age 66.8) treated at 884 hospitals. Overall, 71.8% had no recorded complications, whereas 7.6%, 10.8%, 9.0%, 0.6%, and 0.2% had postoperative complications of CD grades I–V, respectively. The median (25th–75th percentiles) hospital costs were $17.3 K (16.1–19.3) for the no-complications group, and $19.1 K (17.3–22.2), $21.0 K (18.5–25.0), $27.4 K (22.4–33.9), $41.8 K (291–618), and $22.7 K (183–421) for the CD grades I–V complication groups, respectively. The multivariable model identified that complications of CD grades I–V were associated with 11%, 21%, 61%, 142%, and 70% increases in in-hospital costs compared with no complications.

**Conclusions:**

Postoperative complications and their severity are strongly associated with increased hospital costs and health-care resource utilization. Implementing strategies to prevent postoperative complications will improve patients’ clinical outcomes and reduce hospital care costs substantially.

**Supplementary Information:**

The online version contains supplementary material available at 10.1007/s00595-022-02523-6.

## Introduction

Increasing health-care costs are a pressing issue for many developed countries. With primary drivers such as increasing treatment intensity, the technological development of new treatments, and an aging population [1–3], governments and funders such as Medicare and Medicaid Services and insurance companies are continually searching for ways to improve the efficiency of healthcare and reduce its costs [4–7]. Apart from their obvious detriment to patient health, postoperative complications are cited as one of the primary causes of the escalating costs of surgical care. However, there are few accurate reports on the economic impact of postoperative complications.

Calculating the costs of postoperative complications is difficult, because the costs of care vary with the patient’s status. For example, a patient with chronic kidney disease or on maintenance dialysis will require more costly postoperative care [8], and aged patients tend to require more time to recover from surgery than younger patients [9]. Another major issue, as described by Llamas and Ramia [10], is that postoperative complications are seldom recorded with the accuracy and granularity required for their detailed evaluation. This is especially important when evaluating major, but uncommon complications, which require a large database for their assessment. Furthermore, in situations such as in the USA, where charges may vary for the same treatment depending on the insurance plan the patient is on, the assessment of “cost of care” for the complication can be challenging [11].

The aim of this study was to evaluate the costs associated with postoperative complications, using the nationwide clinical registry in Japan, which is linked to a claims database, in a target group of patients undergoing lower anterior resection (LAR). Using the two linked databases, we identified the patients’ pre- and postoperative status, calculated the cost of their hospitalization, and categorized the costs according to the type of health-care resource.

## Methods

### Data source

We collected information from the gastroenterological surgery section of the National Clinical Database (NCD) for this study. The NCD is a platform for nationwide registries in Japan and the gastroenterological section is governed by the Japanese Society for Gastroenterological Surgery (JSGS). The database collects information on all patients undergoing gastroenterological surgery in Japan, and is used for the purpose of certifying surgeons [12, 13]. For specified procedures, detailed information on patients’ pre-, intra-, and postoperative status is collected. The registry conducts yearly audits of the validity of the data, which confirm high accuracy [14, 15].

The NCD also collects claims data from participating hospitals on a voluntary basis. The hospital claims data, called Diagnostics and Procedural Combination (DPC) data, provided by the participating hospitals include information on surgical and anesthetic procedures, drugs, and medical supplies used to treat the patients, and their fees. Data on accommodation fees (hospital fees), intensive-care unit (ICU) care, meals, and rehabilitation are also included. The prices of these procedures/equipment/materials, reflective of their cost, are unified based on the national item-by-item fee for service schedule by the Ministry of Health, Labour, and Welfare, with a strict ban on extra/discounted charges [11]. The fact that all hospitals participating in the DPC reimbursement system are mandated to produce this detailed per item record enables us to estimate the cost of hospitalization [16, 17].

The NCD collected DPC data from approximately 1100 facilities throughout Japan for the fiscal years 2015–2016 (between April, 2015 and March, 2017). Because DPC data and NCD data do not have common personal identifiers, the data were linked using the patients’ sex, age, procedure type, and procedure date for each facility. Patients with more than one possible corresponding case were excluded during the linkage process. The proportion of successful linkages ranged from 88 to 92%, depending on the procedure, and 89.1% of the LAR patients treated at the participating facilities were successfully linked to individual DPC data.

### Patients

From the gastroenterology surgery registry, we selected patients who underwent LAR surgery for rectal cancer between April, 2015 and March, 2017. Patients who had undergone surgery for diseases other than rectal cancer, those with metastatic cancer, those aged < 15 years, those who underwent emergency surgery, those who underwent concomitant procedures for liver resection, gastric cancer, esophageal resection, or surgery on other organs, and those with acute diffuse peritonitis were excluded from the analysis. The study was approved by the Institutional Review Board of The University of Tokyo (11467).

### Outcome of interest

Our primary outcome of interest was the cost of hospitalization. This was calculated by adding all the fees for procedures, medications and medical supplies used, fees for meals, beds (including hospital fees), laboratory testing and imaging, and ICU care recorded in the DPC data. We report the cost in US dollars using a conversion rate of US$1 = 100 Japanese yen. This conversion rate was used to simplify the calculation, although the actual rate was around US$1 = 103 Japanese yen on 1 January, 2021. We also evaluated the postoperative length of stay (poLOS) as a secondary outcome.

### Postoperative complications

The registry collects information on the degree of overall complications that occur within 30 days of surgery, evaluated with the Clavien–Dindo classification. [18] The registry also collects the incidence of individual postoperative complication types, such as surgical-site infection (SSI), anastomotic leakage, abdominal abscess, pneumonia, and paralytic ileus.

### Patients’ characteristics

The patients’ demographic data, preoperative status, and comorbidities were extracted from the database. The information included age, sex, smoking status, body mass index (BMI), preoperative chemotherapy, presence of dyspnea, diabetes mellitus, past diagnosis of coronary artery disease (CAD), maintenance dialysis, heart failure, cerebrovascular disease, ascites, long-term steroid use, weight loss, hemorrhagic tendency, American Society of Anesthesiologist performance status (ASA-PS), activities of daily living (ADL) dependence, and serum creatinine levels. We also collected information on laparoscopy and surgical TNM staging, based on the Union for International Cancer Control version 7, and estimated the average yearly LAR case volume at each participating facility.

### Categorization of hospital costs

We categorized the hospital costs for the care provided between postoperative day 1 and discharge into the costs of surgical care, medications and intravenous fluids, meals, nonsurgical treatments and procedures, hospital beds, ICU care, and others. We assessed how the composition of these cost components varied according to the level of complication, classified based on CD grade, by estimating the average per-patient cost for each category.

### Statistical analysis

We tabulated the characteristics of the whole cohort and estimated the incidence of each complication type, and of complications categorized according to the CD classification. We present the median and 25th–75th percentiles of the hospitalization costs and poLOS according to the CD classification. We constructed two-level hierarchical log-link gamma regression models with random intercepts for the hospitals and fixed effects for all preoperative characteristics, the hospital case volume, and the CD classification for the outcomes of hospitalization cost and poLOS.

In addition, we repeated the descriptive analysis and the regression analysis of hospital costs, restricting them to those incurred on and after postoperative day 1. As subgroup analyses, the regression analysis of full hospital costs was repeated for patients with diabetes mellitus and for patients aged ≥ 75 years, to assess how the effect of complications on in-hospital costs was influenced by these factors. Finally, we employed the model developed using the full cohort to estimate the total hospitalization costs for patients with complications had they not suffered these complications, according to the CD grade. We subtracted this number from the observed cost to calculate the amount that could be saved by preventing these complications.

## Results

**Fig. 1:**
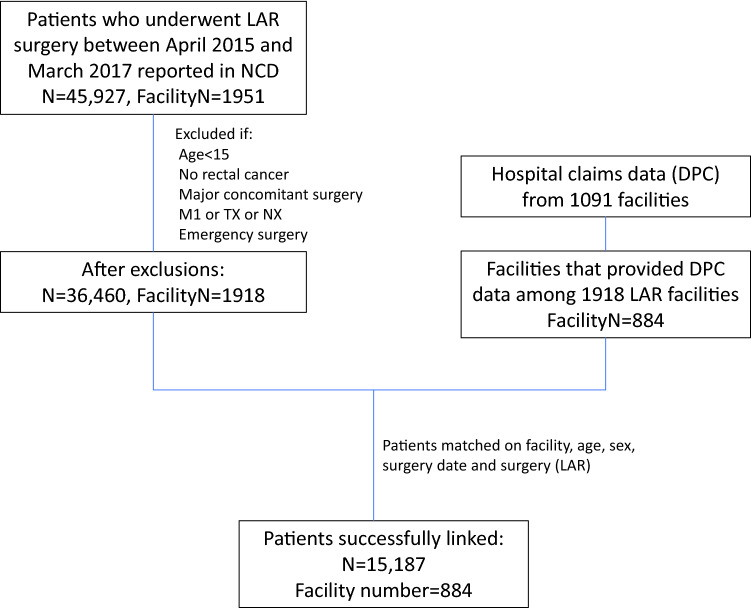
Flow diagram of the patient selection

We identified 45,927 patients who underwent LAR at 1951 facilities between April, 2015 and March, 2017 (Fig. 1). After applying the exclusion criteria, there were 36,460 patients from 1918 facilities. Among these facilities, 884 had provided DPC data for linkage either for fiscal years 2015 or 2016, or both. After linkage, we identified 15,178 patients at these 884 facilities. The mean age of the patients was 66.8 years (± 11.1 years) and 34.1% were women (Table 1). Among these patients, 71.3% underwent a laparoscopy-assisted LAR procedure, 18.9% had diabetes mellitus, 4.0% had a history of CAD, and 0.6% were on maintenance hemodialysis. ADL dependence was recorded in 3.1% of patients. The T stage was Tis (carcinoma in situ) in 303 (2.0%) patients, T1 in 2629 (17.3%) patients, T2 in 3095 (20.4%) patients, and T3 in 7310 (48.1%) patients. The N stage was N0 in 9527 (62.7%) patients, N1 in 3896 (25.7%) patients, and N2 in 1764 (11.6%) patients. Among the 884 facilities analyzed, around 40% had a yearly average LAR case load of < 10, whereas 33.7% had an average case load of ≥ 20.

**Table 1:** Clinical characteristics of 15,187 patients who underwent lower anterior resection for rectal cancer between April, 2015 and December, 2016

Variable	*n*	%
*N*	15,187	
Female	5176	34.1%
Age category (years)		
< 65	5627	37.1%
< 75	5752	37.9%
< 85	3211	21.1%
≥ 85	597	3.9%
BMI category (kg/m^2^)		
< 18.5	1668	11.0%
18.5 to < 25	10,084	66.4%
25 to < 30	2999	19.7%
≥ 30	436	2.9%
Smoker	3499	23.0%
Weight loss ≥ 10%	321	2.1%
ADL dependence	473	3.1%
ASA-PS ≥ 3	1547	10.2%
Comorbidities		
Diabetes mellitus	2866	18.9%
Congestive heart failure	76	0.5%
Coronary artery disease	601	4.0%
Cerebrovascular disease	414	2.7%
Hemodialysis	84	0.6%
Bleeding complication	533	3.5%
Serum creatinine ≥ 2.0 mg/dL	217	1.4%
Long-term steroid use	134	0.9%
Ascites	127	0.8%
T classification		
Tis	303	2.0%
T1	2629	17.3%
T2	3095	20.4%
T3	7310	48.1%
T4	1850	12.2%
N classification		
N0	9527	62.7%
N1	3896	25.7%
N2	1764	11.6%
Laparoscopic procedure	10,824	71.3%
Total number of facilities	884	
Average yearly lower anterior resection volume		
< 10	353	39.9%
10–19	233	26.4%
20–49	237	26.8%
≥ 50	61	6.9%

*BMI* body mass index, *ADL* activities of daily living, *ASA-PS* American Society of Anesthesiologists Physical Status

Supplementary Table 1 summarizes the characteristics of the patients according to their grade of complication. We did not identify a clear association between age and the severity of complications, or between BMI and the severity of complications. However, there were clear associations between preoperative weight loss, ADL dependence, ASA-PS, and the severity of complications. Comorbidities were generally more frequent among patients who suffered severe complications of CD grades III and IV.

Overall, 10,897 (71.8%) patients had no recorded complications, whereas 1149 (7.6%), 1639 (10.8%), 1373 (9.0%), 97 (0.6%), and 32 (0.2%) had recorded postoperative complications of CD grades I–V, respectively (Table2). Supplementary Fig. 1 shows the overall distribution of hospital costs and poLOS. The median [25th–75th percentile] hospital cost was $17.3 K [161–193] for the no-complication group, and $19.1 K [173–222], $21.0 K [185–250], $27.4 K [224–339], $41.8 K [291–618], and $22.7 K [183–421] for the groups with CD grades I–V, respectively. Similarly, the median [25th–75th percentiles] poLOSs were 13 days [10–17], 18 days [13–25], 23 days [16–33], 35 days [24–49], 50 days [33–74], and 10.5 days [4.5–21.5] for the six groups, respectively; noting that CD grade V means the patient died from the complication (Table 2). As these included patients who died early after the surgery, their poLOS may have been shorter than that of patients without complications. When we limited the costs to those incurred on or after postoperative day 1, the hospitalization costs were smaller, but with greater variability, ranging from $3.8 K [3.1–5.0] for no complications to $28.2 K [14.6–42.7] for CD grade IV complications (Supplemental Table 2).

**Table 2:** Incidence of postoperative complications based on the Clavien–Dindo (CD) classification, with associated hospital costs and postoperative length of stay

CD Grade	Frequency, *n*	Frequency, %	Hospital costs*	poLOS
			Median (25th–75th percentiles), $1000	Median (25th–75th percentiles), days
No complication	10,897	71.8%	17.3 (16.1–19.3)	13 (10–17)
CD grade I	1149	7.6%	19.1 (17.3–22.2)	18 (13–25)
CD grade II	1639	10.8%	21.0 (18.5–25.0)	23 (16–33)
CD grade III	1373	9.0%	27.4 (22.4–33.9)	35 (24–49)
CD grade IV	97	0.6%	41.8 (29.1–61.8)	50 (33–74)
CD grade V	32	0.2%	22.7 (18.3–42.1)	10.5 (4.5–21.5)

Complication type	Frequency	%	Hospital cost*	poLOS
			Median (25th–75th percentiles), $1000	Median (25th–75th percentiles), days
Superficial SSI with no other recorded complication	175	1.2%	18.7 (17.2–21.9)	17 (13–24)
Anastomotic leakage	1405	9.3%	28.4 (23.1–33.5)	37 (27–51)
Organ abscess	241	1.6%	28.4 (22.8–35.7)	39 (28–56)
Pneumonia	122	0.8%	29.2 (21.5–45.2)	32.5 (20–54)
Paralytic ileus	463	3.1%	21.9 (19.0–27.2)	25 (18–36)

*poLOS* postoperative length of stay, *CD* Clavien–Dindo, *SSI* surgical site infection

*Hospital costs include costs incurred before surgery, on the day of surgery, and after surgery until discharge

When the costs associated with individual complication types were considered, superficial SSI without any other complication was associated with a small increase in the hospital cost (median [25th–75th percentiles]: $18.7 K [17.2–21.9]) and a modest increase in poLOS (median [25th–75th percentiles]: 17 [13–24]). Anastomotic leakage and organ abscess were associated with similarly large increases in hospital costs (median [25th–75th percentiles]: $28.4 K [23.1–33.5] and $28.4 K [22.8–35.7], respectively), and poLOS (median [25th–75th percentiles]: 37 days [27–51] and 39 days [28–56], respectively). Paralytic ileus was associated with moderate increases in hospital costs (median [25th–75th percentiles]: $21.9 K [19.0–27.2]) and poLOS (25 days [18–36]).

**Fig. 2:**
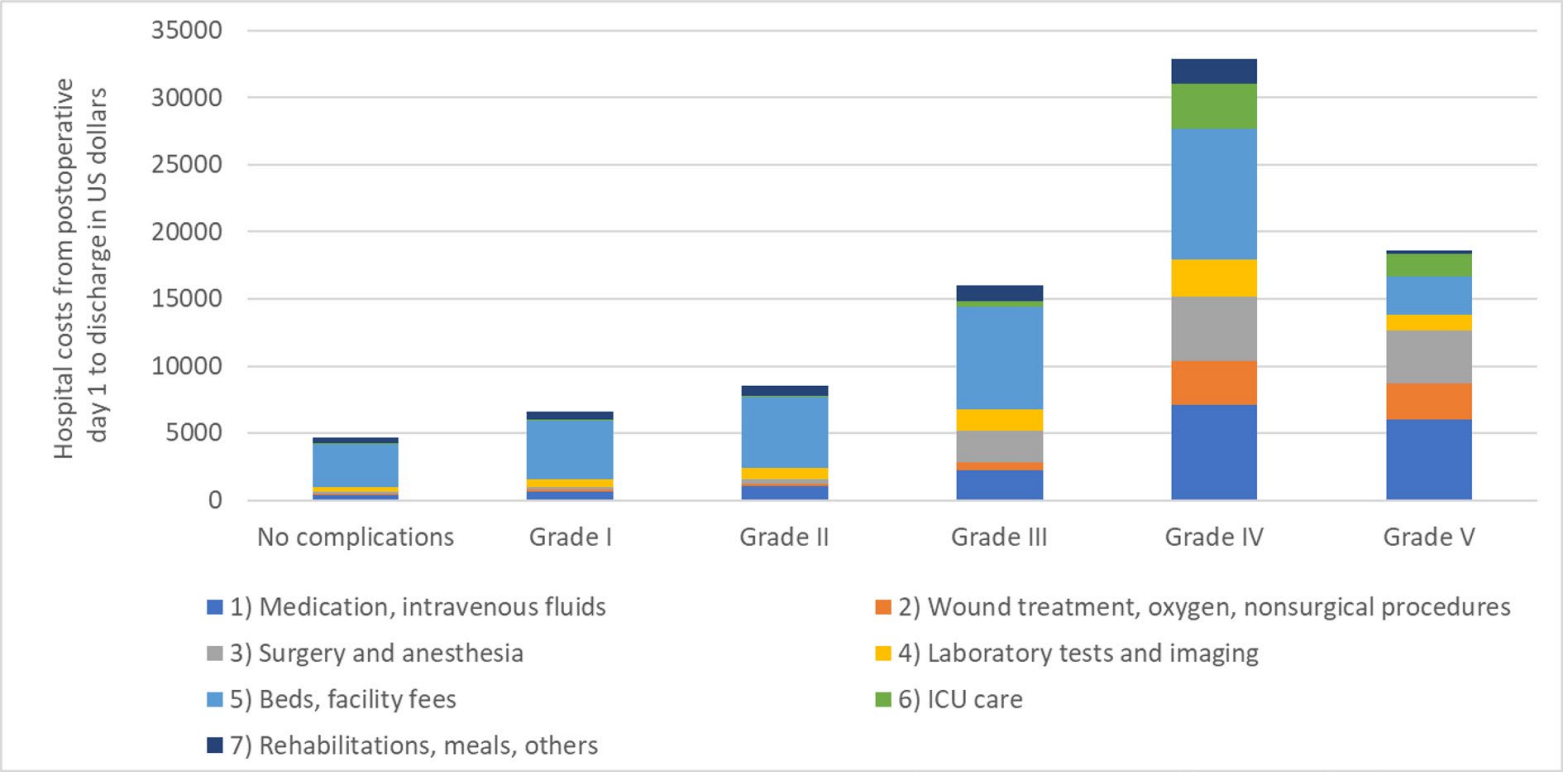
Average costs per patient for in-hospital care from postoperative day 1 to discharge by cost category and CD grading

Most of the average post-surgery hospital costs for patients without complications included fees for beds (including facility fees) (Fig. 2). The composition of the post-surgery hospital costs changed only slightly for patients with grade I or grade II complications, with small increases in costs for the medications and intravenous fluids, laboratory tests, and imaging fees. Complications ≥ grade III were associated with large increases in the proportions of fees for surgery and anesthesia, medications and intravenous fluids, wound treatment, and nonsurgical procedures; grade IV and V complications also required fees for ICU care.

After adjustments were made for patients’ baseline characteristics, comorbidities, tumor stage, as well as laparoscopy and the facility case volume, complications of CD grades I–V were associated with increases in hospital costs of 11% (95% confidence interval [CI]): 10–13), 21% (95% CI 21–23), 61% (95% CI 59–53), 142% (95% CI 131–153), and 70% (95% CI 57–84), respectively (Table 3). When we limited the costs to those incurred on or after postoperative day 1, the relative increases were greater, at 39% (35–43), 77% (72–82), 229% (320–338), 524% (454–590), and 260% (201–330) for CD grades I–V, respectively (Supplementary Table 2).

**Table 3:** Estimates of hospital costs and postoperative length of stay from hierarchical gamma regression models

CD grade	Hospital cost*	poLOS
	Exp (coefficients)	Exp (coefficients)
Overall		
No complications	Reference	Reference
CD grade I	1.11 (1.10–1.13)	1.41 (1.37–1.45)
CD grade II	1.21 (1.21–1.23)	1.77 (1.73–1.81)
CD grade III	1.61 (1.59–1.63)	2.67 (2.60–2.74)
CD grade IV	2.42 (2.31–2.53)	3.40 (3.11–3.71)
CD grade V	1.70 (1.57–1.84)	0.88 (0.75–1.04)

Among diabetes mellitus patients		
No complications	Reference	Reference
CD grade I	1.12 (1.08–1.15)	1.43 (1.34–1.52)
CD grade II	1.20 (1.17–1.23)	1.77 (1.68–1.87)
CD grade III	1.60 (1.55–1.65)	2.76 (2.60–2.92)
CD grade IV	2.51 (2.30–2.74)	3.67 (3.09–4.35)
CD grade V	1.50 (1.19–1.90)	0.43 (0.25–0.75)

Among patients aged ≥ 75 years		
No complications	Reference	Reference
CD grade I	1.09 (1.06–1.12)	1.33 (1.26–1.41)
CD grade II	1.21 (1.18–1.24)	1.70 (1.62–1.79)
CD grade III	1.66 (1.61–1.71)	2.71 (2.56–2.87)
CD grade IV	2.45 (2.24–2.68)	3.31 (2.80–3.93)
CD grade V	1.44 (1.29–1.60)	0.68 (0.55–0.84)

*poLOS* postoperative length of stay, *CD* Clavien–Dindo

*Hospital costs include costs incurred preoperatively, on the day of operation, and postoperatively until discharge

The relative increases in poLOS were more similar to the post-surgery hospital cost than to the full hospital cost, at 41% (95% CI 37–45) for grade I, 77% (95% CI 73–81) for grade II, 167% (160–174) for grade III, 240% (95% CI 211–271) for grade IV, and − 12% (95% CI  − 25–4) for grade V. These relative increases were similar among patients with diabetes mellitus and patients aged ≥ 75 years, although they were slightly less than those for the whole cohort in the latter subgroup.

Among the complication groups classified by CD grade, the CD grade III group had the highest observed total hospital costs ($41.8 M) followed by the CD grade II group ($37.5 M) (Table 4). The difference between the observed costs and the predicted costs had no complication occurred reflects the potential reduction in cost that could be achieved by preventing the complications. This was also highest in the CD grade III group ($16.2 M) followed by the CD grade II group ($6.8 M), accounting for 56.4% and 23.8% of the total potential cost reduction when all complications were prevented.

**Table 4:** Observed hospital costs, predicted costs had complications not occurred, and their differences according to the Clavien–Dindo (CD) classification

CD Classification	*N*	a) Observed total hospital costs, $1000	b) Predicted total hospital cost without complications, $1000	c) Difference (a − b), $1000	% of total cost (%)
CD grade I	1149	23,783	21,296	2,487	8.6
CD grade II	1639	37,481	30,635	6,846	23.8
CD grade III	1373	41,802	25,584	16,218	56.4
CD grade IV	97	4,662	1,905	2,756	9.6
CD grade V	32	1,081	635	446	1.6
All complications	4290	108,809	80,055	28,754	100

*CD* Clavien–Dindo

## Discussion

By collecting data from a Japanese nationwide gastroenterology surgery registry linked to a hospital-based claims database, we quantified the increase in the cost of hospital care associated with postoperative complications, classified with the CD grading system, for patients undergoing LAR surgery. After adjusting for baseline characteristics, comorbidities, tumor stage, laparoscopy, and facility case volume, complications of CD grades I–V were associated with 11%, 21%, 61% 142%, and 70% increases in hospital costs, respectively. For patients without complications and those with grade I–II complications, the postoperative hospital costs consisted mainly of fees for hospital beds (facility fees). Fees for surgery, anesthesia, medications, tests, imaging, and ICU care were more prevalent for patients with CD grade ≥ 3 complications.

One of the challenges in accurately assessing the impact of postoperative complications on hospital costs lies in their correct identification. As noted previously, [10, 19] the definition and reporting of complications may vary by practice and region, and their accuracy may not be optimal, especially for lower-grade complications. This is especially true for studies that rely solely on claims databases to identify complications, [20] but it is also relevant for registry-based studies, as suggested by past validation studies. [14] Under-capture of low-grade complications may result in patients with minor complications being included in the no-complication group and lead to underestimation of the impact of these complications.

The ability to classify complications by their severity is important, because the cost of hospital care for patients with complications varies greatly according to the severity of the complication, as demonstrated by our study and others [19, 21, 22]. Our results are consistent with those of other studies that have found small to modest increases in hospital costs for grade I and II complications and much greater increases for grade III and IV complications [19, 22, 23]. However, the possible effect modifications by factors such as age and baseline comorbidities have not been assessed extensively. In the present study, the impact of complications on hospital costs or poLOS did not differ substantially between either the subgroup of patients aged ≥ 75 years or those with a baseline diabetes mellitus diagnosis and the other patients.

In our cohort, the increases in hospital costs arising from lower-grade (CD I and II) complications were attributable mainly to increases in hospital bed fees, reflecting the extended poLOS in these groups. For complications of grades III and IV, although bed fees remained a major cost component, fees for treatments such as additional surgery, medications, tests, and imaging became more prominent. Due to both the high frequency and the size of the increased cost, CD grade III complications were responsible for the largest proportion of the estimated total increase in hospital costs resulting from complications, accounting for 56.4% of the total increase. This finding differs from that reported by Cosic et al. [21]. The majority of their liver resection patients had CD grades I and II complications (18% and 43% of the cohort, respectively), and grade II complications were associated with a greater increase in cost ($8000). Further research is required to evaluate how different procedures influence the impact of complications on hospital cost.

Precise evaluation of the economic impact of postoperative complications allows for improved planning of management strategies, both at the facility level and at the community level. Japan employs the diagnostic and procedure combination per-diem payment system (DPC/PDPS) which is a flat-rate per-diem reimbursement system based on the diagnosis for hospitalizations [24]. The per-diem fee for hospitalization is reduced when the length of stay reaches certain thresholds; for example, on 15^th^ and 30^th^ day for rectal cancer patients undergoing LAR in 2016, and although some complications are compensated for, the occurrence of complications will generally reduce hospitals’ profit. Quantification of the financial burden of postoperative complications will allow us to also quantify the financial benefits of preventive measures that may at times seem costly. The financial incentives for hospitals to minimize postoperative complications may be even stronger in nations employing different payment systems, such as diagnosis-related group (DRG) [25, 26] or global budgeting [27, 28].

This study had some limitations. First, we were unable to fully assess the effect on costs of the individual complication types, because our database does not capture the chronological order of adverse events, and therefore, we did not know whether the events were independent or sequelae. As the development of a complication is a strong risk factor for the development of another [29] we could not assess the cost impact of a type of complication without knowing its relationship to other events. Second, the data were taken from a representative nationwide database in Japan, but participation in the DPC linkage study was voluntary and incomplete. The hospitals that declined to participate may have had distinct characteristics, and our findings may not be generalizable to those facilities. Moreover, healthcare costs are highly dependent on the insurance schemes of the nation or on the plan in which a patient is enrolled. Notably, Japanese patients have a characteristically long length of stay for both surgical and non-surgical hospitalizations [16, 30, 31]. Our findings may not be transferable to other countries, where the fee schedules and practice patterns are substantially different. Third, we were able to assess only the costs incurred during hospitalization. Further research is required to understand the impact of complications on healthcare costs, including the costs of out-patient follow-up care. Finally, as this was an observational study based on a clinical registry, residual confounding by factors associated with postoperative complications and LOS are possible. We adjusted for important clinical factors such as age and comorbidities collected in the registry, but other factors such as frailty, nutritional status, or socio-economic factors may influence our estimation.

## Conclusions

Postoperative complications and their severity are strongly associated with increases in hospital costs, the utilization of health-care resources, and poLOS. Increased measures to prevent postoperative complications will not only improve patients’ clinical outcomes, but substantially reduce the cost of healthcare.

## Supplementary Information

Below is the link to the electronic supplementary material.

Supplementary file1 (DOCX 79 kb)

## Declarations

### Approval of the research protocol

The study was reviewed and approved by the Institutional Review Board of the University of Tokyo Graduate School of Medicine and Faculty of Medicine (ID: 11467).

### Registry and the Registration No. of the study/Trial

NA.

### Animal studies

NA.
