# Supplementary material for: Cost of postoperative complications of lower anterior resection for rectal cancer: a nationwide registry study of 15,187 patients
Source: Surg Today. 2022 May 24;52(12):1766–74. doi: 10.1007/s00595-022-02523-6 (PMC9700610; doi:10.1007/s00595-022-02523-6)
Supplement: Supplementary file 1 — Supplementary file1 (DOCX 79 kb) [file 595_2022_2523_MOESM1_ESM.docx]

Supplementary Material

Supplementary Table 1.

Characteristics of patients by the Clavien–Dindo classification

Supplementary Table 2.

Hospital Cost occurring on and after postoperative day 1 according to postoperative complications based on the Clavien–Dindo (CD) classification, and the estimates from the hierarchical gamma regression model

Supplementary Figure 1.

Distribution of hospital costs and postoperative length of stay for patients who underwent Lower Anterior Resection between April 2015 and March 2017

Supplementary Table 1. Characteristics of patients by the Clavien–Dindo classification

|  |  | No complications |  | Grade I |  | Grade II |  | Grade III |  | Grade IV |  | Grade V |  |
| --- | --- | --- | --- | --- | --- | --- | --- | --- | --- | --- | --- | --- | --- |
| N |  | 10,897 |  | 1149 |  | 1639 |  | 1373 |  | 97 |  | 32 |  |
| Female |  | 4107 | 37.7% | 353 | 30.7% | 412 | 25.1% | 273 | 19.9% | 17 | 17.5% | 14 | 43.8% |
| Age category (years) | < 65 | 4052 | 37.2% | 417 | 36.3% | 572 | 34.9% | 551 | 40.1% | 29 | 29.9% | 6 | 18.8% |
|  | 65-74 | 4094 | 37.6% | 406 | 35.3% | 672 | 41.0% | 537 | 39.1% | 38 | 39.2% | 5 | 15.6% |
|  | 75-84 | 2324 | 21.3% | 269 | 23.4% | 334 | 20.4% | 245 | 17.8% | 24 | 24.7% | 15 | 46.9% |
|  | 85 and above | 427 | 3.9% | 57 | 5.0% | 61 | 3.7% | 40 | 2.9% | 6 | 6.2% | 6 | 18.8% |
| BMI category (kg/m^2^) | < 18.5 | 1200 | 11.0% | 129 | 11.2% | 168 | 10.3% | 156 | 11.4% | 11 | 11.3% | 4 | 12.5% |
|  | 18.5 to < 25 | 7227 | 66.3% | 758 | 66.0% | 1088 | 66.4% | 928 | 67.6% | 64 | 66.0% | 19 | 59.4% |
|  | 25 to < 30 | 2150 | 19.7% | 228 | 19.8% | 341 | 20.8% | 256 | 18.6% | 19 | 19.6% | 5 | 15.6% |
|  | ≥ 30 | 320 | 2.9% | 34 | 3.0% | 42 | 2.6% | 33 | 2.4% | 3 | 3.1% | 4 | 12.5% |
| Smoker |  | 2339 | 21.5% | 287 | 25.0% | 419 | 25.6% | 416 | 30.3% | 33 | 34.0% | 5 | 15.6% |
| Weight loss ≥ 10% | | 185 | 1.7% | 34 | 3.0% | 55 | 3.4% | 41 | 3.0% | 5 | 5.2% | 1 | 3.1% |
| ADL dependence | | 293 | 2.7% | 45 | 3.9% | 59 | 3.6% | 60 | 4.4% | 10 | 10.3% | 6 | 18.8% |
| ASA-PS ≥ 3 | | 1012 | 9.3% | 130 | 11.3% | 219 | 13.4% | 158 | 11.5% | 20 | 20.6% | 8 | 25.0% |
| Comorbidities | |  |  |  |  |  |  |  |  |  |  |  |  |
|  | Dialysis Mellitus | 1950 | 17.9% | 237 | 20.6% | 364 | 22.2% | 282 | 20.5% | 29 | 29.9% | 4 | 12.5% |
|  | Congestive heart failure | 45 | 0.4% | 9 | 0.8% | 9 | 0.5% | 11 | 0.8% | 2 | 2.1% | 0 | 0.0% |
|  | Coronary artery disease | 407 | 3.7% | 54 | 4.7% | 71 | 4.3% | 58 | 4.2% | 9 | 9.3% | 2 | 6.3% |
|  | Cerebrovascular disease | 261 | 2.4% | 38 | 3.3% | 59 | 3.6% | 48 | 3.5% | 6 | 6.2% | 2 | 6.3% |
|  | Hemodialysis | 50 | 0.5% | 6 | 0.5% | 11 | 0.7% | 14 | 1.0% | 3 | 3.1% | 0 | 0.0% |
|  | Bleeding complication | 365 | 3.3% | 38 | 3.3% | 63 | 3.8% | 58 | 4.2% | 8 | 8.2% | 1 | 3.1% |
|  | Creatinine ≥ 2.0mg/dL | 129 | 1.2% | 17 | 1.5% | 32 | 2.0% | 29 | 2.1% | 7 | 7.2% | 3 | 9.4% |
| Long-term steroid use | | 85 | 0.8% | 11 | 1.0% | 19 | 1.2% | 16 | 1.2% | 2 | 2.1% | 1 | 3.1% |
| Ascites |  | 89 | 0.8% | 10 | 0.9% | 14 | 0.9% | 12 | 0.9% | 1 | 1.0% | 1 | 3.1% |
| T classification | |  |  |  |  |  |  |  |  |  |  |  |  |
|  | Tis | 216 | 2.0% | 29 | 2.5% | 38 | 2.3% | 18 | 1.3% | 1 | 1.0% | 1 | 3.1% |
|  | T1 | 2017 | 18.5% | 188 | 16.4% | 216 | 13.2% | 193 | 14.1% | 11 | 11.3% | 4 | 12.5% |
|  | T2 | 2281 | 20.9% | 213 | 18.5% | 323 | 19.7% | 253 | 18.4% | 21 | 21.6% | 4 | 12.5% |
|  | T3 | 5108 | 46.9% | 574 | 50.0% | 848 | 51.7% | 707 | 51.5% | 52 | 53.6% | 21 | 65.6% |
|  | T4 | 1275 | 11.7% | 145 | 12.6% | 214 | 13.1% | 202 | 14.7% | 12 | 12.4% | 2 | 6.3% |
| N classification | |  |  |  |  |  |  |  |  |  |  |  |  |
|  | N0 | 6935 | 63.6% | 724 | 63.0% | 969 | 59.1% | 821 | 59.8% | 58 | 59.8% | 20 | 62.5% |
|  | N1 | 2770 | 25.4% | 271 | 23.6% | 466 | 28.4% | 359 | 26.1% | 20 | 20.6% | 10 | 31.3% |
|  | N2 | 1192 | 10.9% | 154 | 13.4% | 204 | 12.4% | 193 | 14.1% | 19 | 19.6% | 2 | 6.3% |
| Laparoscopic procedure | | 7886 | 72.4% | 768 | 66.8% | 1123 | 68.5% | 969 | 70.6% | 59 | 60.8% | 19 | 59.4% |

Abbreviations: BMI: Body mass index, ADL: Activities of daily living, ASA-PS: American Society of Anesthesiologists Physical Status

Supplementary Table 2. Hospital costs occurring on and after postoperative day 1 according to postoperative complications based on the Clavien–Dindo (CD) classification, and the estimates made with the hierarchical gamma regression model

| CD Grade | Frequency,  n | Frequency,  % | Hospital costs*: | Hospital costs*: |
| --- | --- | --- | --- | --- |
|  |  |  | median (25^th^–75^th^ percentiles), $1000 | Exp(coefficients) |
| No complication | 10,897 | 71.8% | 3.8 (3.1–5.0) | Reference |
| CD grade I | 1149 | 7.6% | 5.3 (3.9–7.4) | 1.39 (1.35–1.43) |
| CD grade II | 1639 | 10.8% | 6.9 (5.0–10.1) | 1.77 (1.72–1.82) |
| CD grade III | 1373 | 9.0% | 13.1 (8.9–18.9) | 3.29 (3.20–3.38) |
| CD grade IV | 97 | 0.6% | 28.2 (14.6–42.7) | 6.24 (5.65–6.90) |
| CD grade V | 32 | 0.2% | 8.8 (3.3–27.1) | 3.60 (3.01–4.30) |

*Hospital costs are limited to those incurred between postoperative day 1 and discharge


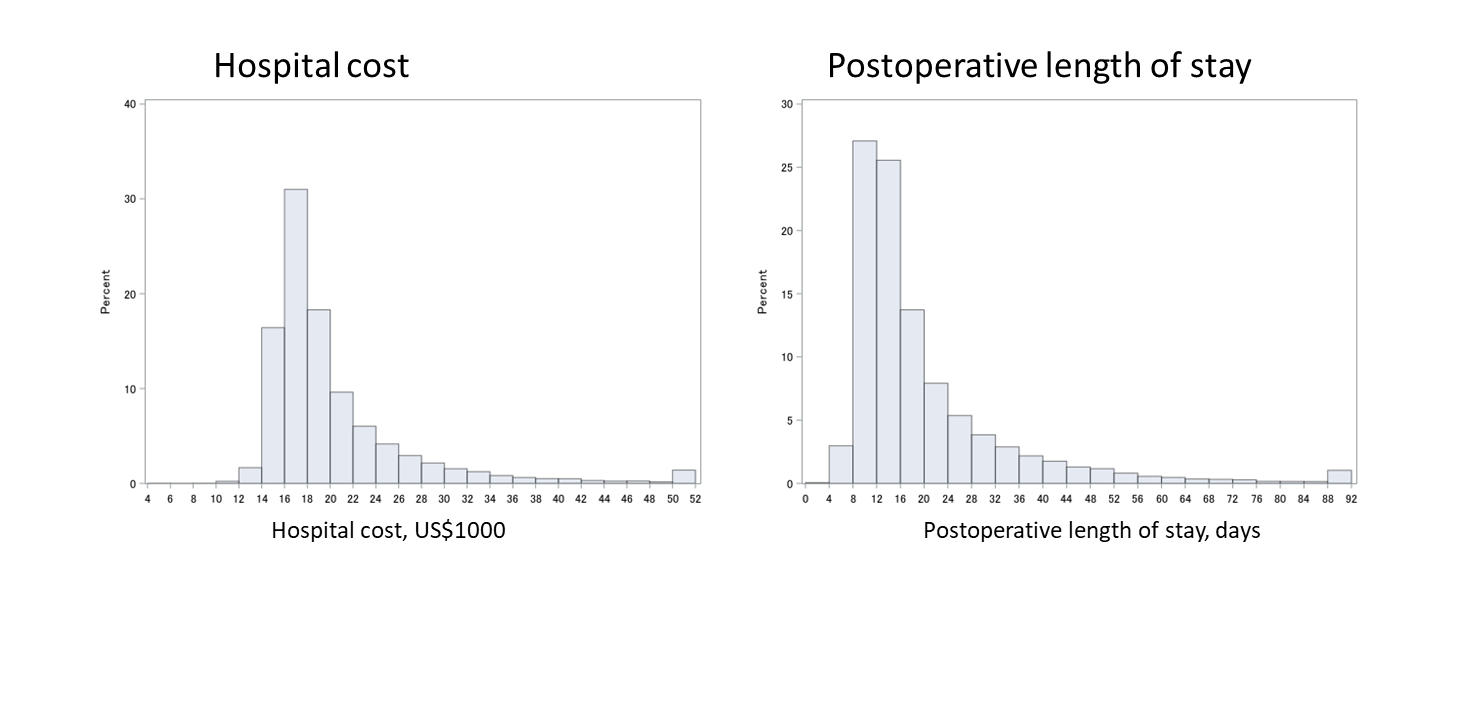


Supplementary Figure 1. Histograms of hospital costs and postoperative length of stay in patients who underwent Lower Anterior Resection between April 2015 and March 2017

*The cost is capped at $50,000, and the postoperative length of stay is capped at 90 days for ease in visualization.
